# Biofilm Formation by *ica*-Negative Ocular Isolates of *Staphylococcus haemolyticus*

**DOI:** 10.3389/fmicb.2018.02687

**Published:** 2018-11-14

**Authors:** Sasmita Panda, Durg Vijai Singh

**Affiliations:** Infectious Disease Biology, Institute of Life Sciences, Bhubaneswar, India

**Keywords:** biofilm, *Staphylococcus haemolyticus*, extracellular DNA, proteins, autolysis, sortase A

## Abstract

*Staphylococcus haemolyticus* is the second most frequently isolated CoNS from ocular infections and human blood cultures. In this study, we examined 18 ocular *S. haemolyticus* isolates for their capacity to form biofilm and conducted detachment assay to determine the composition of the biofilm matrix and involvement of various elements in cell lysis. PCR identified the presence of biofilm-associated genes, and *ica* operon and CLSM visualized the components of the biofilm matrix. We found that PIA-independent biofilm formation is the characteristic feature of *S. haemolyticus* isolates, irrespective of the sources of isolation, and protein or DNA or both are the major components of the biofilm matrix. Cell lysis enabling DNA release was an essential step for biofilm attachment during the initial stages of biofilm development. The *srtA* transcript expression study indicates its role in the early stages of biofilm development. We found the presence of antibiotic resistance genes in the eDNA and gDNA thus suggesting the possible role of biofilm in horizontal gene transfer of antibiotic resistance determinants. The overall study indicates that *S. haemolyticus* formed the biofilm comprising of protein or DNA or both and *srtA* play a role in the initial development of biofilm.

## Introduction

*Staphylococcus haemolyticus* is the second most frequently isolated pathogen from ocular infections and blood cultures among the coagulase-negative staphylococci (CoNS) (Takeuchi et al., 2005; Makki et al., 2011; Becker et al., 2014). This organism is implicated in hospital-acquired infections occurring through implanted medical devices (Mehta and Kumari, 1997; Mack et al., 2006) and exhibits the highest level of multidrug resistance including hetero-resistance to glycopeptides thereby limiting the therapeutic options (Fredheim et al., 2009; Czekaj et al., 2015). Biofilms formed by *S. haemolyticus* further advance its ability to escape the host defense besides rendering antimicrobial resistance (Krzyminska et al., 2015). Hence, to study the mechanism underlying the biofilm formation in *S. haemolyticus* is warranted.

The ability to form biofilm is considered as one of the most important virulence factor (Mack et al., 2006) that has a role to play in various medical implants related chronic infections (Barbieri et al., 2015). Biofilm formation in *Staphylococcus epidermidis* is a two-step process. (i) Initial attachment mediated by many factors like cell wall anchored surface proteins (i.e., Fbe, Bhp) and cell wall lytic enzyme autolysin E (AltE) (Heilmann et al., 1997) and (ii) accumulation phase involving cell proliferation and intercellular adhesion. Both Fbe (Cucarella et al., 2001) and AtlE (Heilmann et al., 1997) bind to the host factors fibrinogen and vitronectin and Bhp contributes to primary attachment as well as in intracellular adhesion (Davis et al., 2001). The role of accumulation-associated protein (Aap), independently or in association with polysaccharide intercellular adhesin (PIA) in biofilm formation was also established (Hussain et al., 1997; Klingenberg et al., 2004; Rohde et al., 2005). The PIA synthesized by *icaRADBC*-encoded protein (Heilmann et al., 1996; Mack et al., 1996), is neither essential nor necessary component for the CoNS biofilm formation (Kogan et al., 2006; Frank and Patel, 2007; Fredheim et al., 2009). DNA, RNA, proteins, and polysaccharides other than PIA could be crucial components of extracellular matrix (ECM) in CoNS biofilm (Hanssen et al., 2004; Sadovskaya et al., 2005; Tormo et al., 2005; Qin et al., 2007; Fredheim et al., 2009).

Unlike *Staphylococcus aureus* and *S. epidermidis*, no much information is available about the virulence of *S. haemolyticus* particularly in the context of ocular infections. The involvement of genes in cell lysis and extracellular DNA (eDNA) release (i.e., *cidABC*, *lrgAB*) and the role of quorum sensing genes and global regulators that are known to participate in biofilm formation in *S. aureus* and *S. epidermidis* (Rice et al., 2007) is not well studied in *S. haemolyticus*. Moreover, the role of eDNA in the exchange of antibiotic resistance genes (ARGs) via horizontal gene transfer (HGT) is also not clear. Cell surface proteins such as fibrinogen binding protein (*fbp*), elastin binding protein (*ebp*), and collagen binding protein (*cbp*) are known to contribute to the *S. aureus* biofilm phenotype (Foster and Hook, 1998; O’Neill et al., 2008). These cell surface proteins contain a C-terminal LPXTG motif recognized by sortase A (*srtA*) which facilitates their adhesion to the cell wall (O’Neill et al., 2008; Cascioferro et al., 2015). Biofilm formation and its propagation is a very well-regulated process (O’Toole et al., 2000). Autolysis and adhesion of surface-associated proteins mediated by *cidA* and *srtA* are crucial for biofilm formation (Rice et al., 2007; Cascioferro et al., 2015). No report showed the temporal role of these processes and genetic components in *S. haemolyticus* biofilm formation of isolates from ocular infections.

We had a cohort of 18 ocular isolates of *S. haemolyticus* and were examined for their capacity to form biofilm and for its composition. Also, we looked for the presence of genetic determinants associated with biofilm formation and ARGs in eDNA. Besides, the effect of autolysis and adhesion of surface-associated proteins was examined to ascertain the role of these processes in biofilm development.

## Materials and Methods

### Ethics Statement

The Institutional Review Board (IRB) of LV Prasad Eye Institute (LEC/08/110/2009) approved the study, and the data were analyzed anonymously and reported.

### Bacterial Strains

Eighteen non-duplicate *S. haemolyticus* strains isolated from patients with a variety of ocular infections at LV Prasad Eye Institute, Bhubaneswar, India, during 2007–2011 and characterized previously following the standard methods (Panda et al., 2014, 2016) were included in the study. These isolates were from patients with microbial keratitis (*n* = 8), nasolacrimal duct obstruction (*n* = 1), blepharitis (*n* = 1), and keratoconjunctivitis (*n* = 1). Also, seven strains were isolated from the asymptomatic healthy conjunctiva. The 16S rDNA sequencing and amplification of species-specific *nuc* gene confirmed the identity of isolates as *S. haemolyticus* (Panda et al., 2016).

### Quantitative Assay of Biofilm

The capacity of biofilm formed by *S. haemolyticus* was determined by the method modified by Fredheim et al. (2009). Briefly, after diluting bacterial cultures to absorbance 0.05 OD at 595 nm, 200 μl of diluted cultures were added to 96 well polystyrene plates (COSTAR; Sigma) and were allowed to grow for 24 h at 37°C statically. The planktonic cells were removed by inverting the plate on the paper towel and washed the plates once with 1× PBS and then stained with 125 μl of 0.1% crystal violet for 15 min at room temperature. The bound dye was dissolved in the ethanol-acetone mixture (70:30) and recorded the absorbance at 540 nm (Thermo Scientific Varioskan, United States). We performed the biofilm assay in a single run of four wells in biological triplicates. Isolates were considered biofilm positive if the strain had an OD of ≥0.25. Biofilm forming capacities of all isolates were determined in TSB/BHIB, TSB/BHIB with 1% glucose (TSB_*glu*_/BHIB_*glu*_) and also in TSB/BHIB with 3% NaCl (TSB_*NaCl*_/BHIB_*NaCl*_). We used *S. epidermidis* ATCC 35984 as a positive control for PIA production and biofilm formation in all the experiments.

### Confocal Laser Scanning Microscopy (CLSM) for Biofilm Estimation

Diluted bacterial cultures grown in TSB_*glu*_ were added to 8 well-chambered slides (Nunc) and allowed to grow statically at 37°C for 24 h. The chambers were inverted, washed with 1× PBS and stained with Acridine Orange (Sigma; 0.02%) for 15 min in the dark at room temperature. The cells were fixed using 10% neutral formalin, kept in the dark for 30 min, then sealed by the coverslip and collected the images by Leica TCS SP5 confocal scanning system (Leica Microsystem, Mannheim, Germany). Excitation at 488 nm detected the fluorescence of Acridine Orange and emission with a 500–530 nm bandpass filter. The z-stacks were collected at 1 μm intervals and exported as.tif files from Leica LAS AF (version 1.8.2) software and imported into the ImageJ (version 1.41) program (USNIH, Bethesda, MD, United States). PHILIP program analyzed the randomly selected five different fields for imaging and quantification of the mean thickness of biofilm.

### Detachment Assay of Biofilm

We performed detachment assays to determine the components of biofilm matrix by the method of Fredheim et al. (2009) using reagent and enzymes (i) 40 mM NaIO_4_ (Sigma; S1878), (ii) 0.1 mg/ml proteinase K (Sigma; P2308), and (iii) 0.5 mg/ml DNase I (Sigma; DN25). Diluted bacterial isolates were seeded to 96 well polystyrene plates (COSTAR; Sigma) and were allowed to grow for 24 h at 37°C under static condition. Then the above reagents were added to individual wells at 0 and 24 h. After incubation, the biofilm was disrupted by mixing and remaining adhered cells were quantified by crystal violet staining by the method described above. *S. epidermidis* ATCC 35984, known to produce PIA dependent polysaccharide matrix, was used as a control.

We performed the detachment assay in duplicate wells in three biological replicates by the method described above (Fredheim et al., 2009). The percent detachment was calculated from the average difference between the treated wells and the control wells and divided the results into three categories: (i) no detachment (<10%), (ii) intermediate detachment (10–50%), and (iii) strong detachment (>50%). *S. epidermidis* ATCC 35984 and SHN65 grown for 24 h on 8 well-chambered slides were subjected to NaIO_4_, proteinase K, and DNase I treatment and allowed to grow for another 24 h at 37°C. Then stained the adherent biofilm comprised of polysaccharides, eDNA, and protein grown in chamber slides with wheat germ agglutinin-Alexa Fluor 488 conjugate (Invitrogen; 1 μg/ml), DAPI (Sigma; 5 μg/ml) and sypro ruby (Invitrogen; 1×) and collected the images by CLSM. WGA-Alexa Fluor 488 and sypro ruby fluorescence detected by excitation at 488 nm, and emission was collected with a 510–570 nm and 600–700 nm bandpass filter, respectively. Excitation at 405 nm detected DAPI fluorescence and emission collected with a 410–475 nm bandpass filter. The z-stacks were collected at 1 μm intervals and exported as.tif files from Leica LAS AF (version 1.8.2) software.

### Time Point Inhibition Assay

We added Proteinase K and DNase I to the wells containing *S. haemolyticus* SHN65 and SH1965 and *S. epidermidis* ATCC 35984 at 0, 1, 2, 3, 4, 6, 8, 10, 12, and 24 h to see the effect on biofilm formation. All wells were incubated for a total of 24 h at 37°C under static condition and the wells treated at 24 h were further allowed for another 12 h after the addition of the reagents.

### Effect of Inhibitor on Biofilm Formation

We determined the inhibitory effect of Sodium polyanethole sulphonate (PAS) on autolysis by the method described earlier (Rice et al., 2007). PAS (0.5 mg/ml) was added to the respective wells at different time points and incubated for a total of 24 h, and the biofilm was allowed to grow for further 12 h after addition of PAS and estimated by the method described above.

### Extraction of Extracellular DNA and Quantification

Extracellular DNA was extracted by the method described by Rice et al. (2007). Briefly, 100 μl of filter-sterilized supernatant from each isolate was transferred into a chilled tube containing 300 μl of TE buffer and extracted once with an equal volume of phenol/chloroform/isoamyl alcohol (25:24:1) by centrifuging at 18,000 × *g* for 20 min at 4°C. The supernatant was further extracted once with an equal volume of chloroform/isoamyl alcohol (24:1). The aqueous phase of each sample was then mixed with 3 volume of ice-cold 100% ethanol and 1/10 volume of 3 M sodium acetate (pH 5.2) and stored at −20°C. The next day, the ethanol precipitated DNA was collected by centrifugation for 20 min at 4°C and 18,000 × *g*, washed with ice-cold 70% ethanol, air dried, and dissolved in 30 μl of nuclease-free water.

The extracted eDNA was diluted 10 times (1:9) using nuclease-free water and used as a template. PCR mixture contained: 4 μl of 5× PCR buffer, 1 μl of 25 mM MgCl_2_, 2 μl each of dNTPs (Promega, Madison, WI, United States), 2 μl each of forward and reverse primer (5 pmol/μl) (GCC Biotech, New Delhi), 0.25 μl of 5 U/μl Taq polymerase (Promega, Madison, WI, United States), 1 μl of diluted eDNA, and 7.75 μl of nuclease-free water. The PCR was programmed as follows: initial denaturation at 94°C for 2 min, followed by 25 cycles consisting of denaturation at 94°C for 30 s, annealing at 52°C for 30 s, extension at 72°C for 30 s, and a final extension at 72°C for 5 min. PCR products were electrophoresed on 1.5% agarose gel, stained with EtBr and photographed using a gel documentation system (Bio-Rad, United States). The images were analyzed using ImageJ software and the eDNA quantified by amplifying 16S rDNA, 23S rDNA, and *gyrA* genes, and taking into account the ratio of released eDNA and biomass of the isolate.

### PCR Assays

PCR determined the presence of *icaA* and *icaD* genes by the method described earlier (Arciola et al., 2001). We used *S. epidermidis* ATCC 35984 as a positive control for *icaA* and *icaD* detection. Also, PCR was used to determine the presence of antibiotic resistance genes among *S. haemolyticus* (Panda et al., 2016). Supplementary Table S1 showed the cyclic conditions of PCR and primers used for the detection of biofilm-associated gene and amplification of ARG. The amplicons of *cidA*, *cidB*, *lrgB*, *sigB*, *mgrA*, *lytR*, *lytS*, *ebpS*, *fbp*, *agrA*, *agrB*, *luxS*, and *srtA* genes were sequenced and submitted to GenBank database under accession numbers KU518895–KU518907.

### RT-PCR Assays

To determine the expression of biofilm-associated genes, the aliquots of 4-h grown culture in TSB_glu_ diluted to an absorbance of 0.05 OD at 595 nm were seeded into 35 mm Petri plate (Tarson) and incubated at 37°C for 4 h under static condition. The adherent cells were scraped using ice chilled resuspension buffer (50 mM Tris–HCl, 10 mM EDTA, 500 mM NaCl, pH 8.0) and centrifuged at 18,000 *g* for 5 min at 4°C. The pellet was used to isolate RNA using Trizol reagent (Invitrogen, United States) according to the manufacturer’s instructions, and synthesized cDNA using 500 ng RNA and SuperScript VILO cDNA synthesis kit (Invitrogen, United States). The cDNA was diluted 30 times with nuclease-free water (1:29), and 1 μl of cDNA was used as a template in RT-PCR. The cyclic conditions were as follows: initial denaturation at 94°C for 2 min followed by 25 cycles consists of denaturation at 94°C for 30 s, annealing at 52°C for 30 s, and extension at 72°C for 30 s. The final extension was at 72°C for 5 min. Similarly, RT-PCR determined the expression of *srtA* in the biofilm of SHN65 grown for 4, 8, 12, 24, 48, and 72 h. Supplementary Table S1 shows the list of primers used in the expression study for biofilm-associated genes.

## Results

### Quantitative Estimation of Biofilm Formation

**FIGURE 1:**
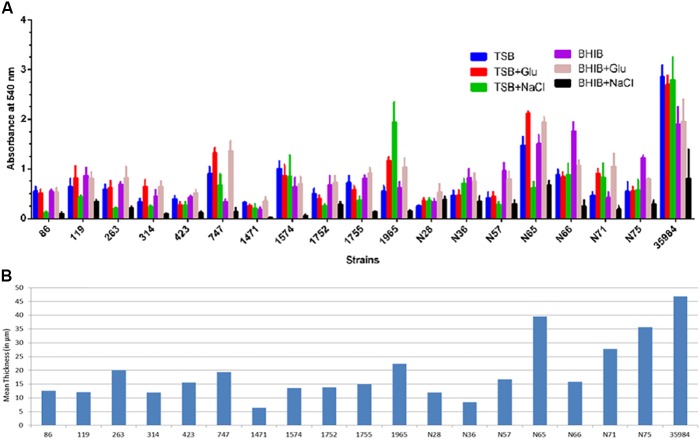
Quantification of biofilm formed by *S. haemolyticus* isolates in 24 h **(A)** crystal violet assay using TSB and BHIB with or without supplementation, **(B)** CLSM analysis represented as mean thickness of biofilm-induced in TSB_*glu*_.

In this study, we checked the isolates for their ability to form biofilm in different media, instead for examining the differences in the rate of biofilm development. We found all the ocular *S. haemolyticus* isolates belonging to the infected eye, and healthy conjunctiva formed a biofilm on polystyrene microtiter plates. The capacity of biofilm formation by strains varied with media used (Figure 1A). In general, all the strains efficiently formed biofilms in both media TSB_glu_ and BHIB_glu_. However, one strain from the infected eye (SH1965) and two isolates from healthy conjunctiva (SHN66 and SHN75) formed better biofilm in TSB_NaCl_ and BHIB, respectively. Isolate SHN65 formed the highest biofilm among the strains tested. The biofilm forming capacity of the isolates was further quantified by estimating the mean thickness of the biofilm formed in TSB_glu_ by CLSM (Figure 1B). Supplementary Figure S1 showed the representative CLSM images of the biofilm by all the ocular isolates.

### Composition of the Biofilm Matrix

**FIGURE 2:**
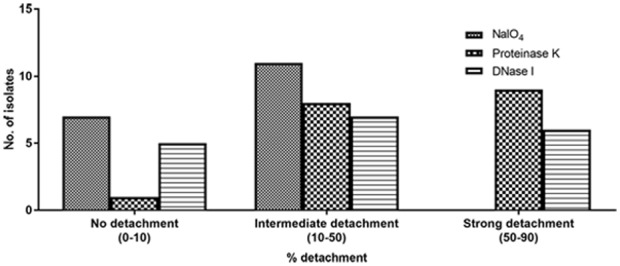
Percent detachment of preformed biofilms obtained with 18 ocular *S. haemolyticus* isolates after treatment with NaIO_4_, proteinase K or DNase I.

All isolates of *S. haemolyticus* showed decreased capacity to form biofilm in the presence of proteinase K and/or DNase I when added at the initial point of inoculation in both TSB_glu_ (Supplementary Figures S2A,B) and BHIB_glu_ (Supplementary Figures S2C,D). These observations thus indicate the role of proteins and eDNA in biofilm development. We then performed detachment assay to see the effect of NaIO_4_, proteinase K and DNase I on mature biofilm in both the media TSB_glu_ (Supplementary Figures S3A–C) and BHIB_glu_ (Supplementary Figures S3D–F). The extent of detachment of biofilm was media independent. We found that 17 of 18 ocular *S. haemolyticus* isolates showed detachment after treatment with proteinase K, and 14 isolates each after treatment with DNase I, and NaIO_4_, respectively. We noticed that none of the strains showed strong detachment (50–90%) upon NaIO_4_ treatment. However, 11 isolates showed moderate (10–50%), and three strains showed low level (0–10%) of detachment, respectively. While studying the effect of proteinase K and DNase I on biofilm detachment, we found 17 isolates showing intermediate and 13 strong detachment, respectively (Figure 2). The positive control *S. epidermidis* ATCC 35984 displayed strong detachment upon NaIO_4_ treatment. Therefore, we selected the best biofilm forming strain SHN65 for the further study.

**FIGURE 3:**
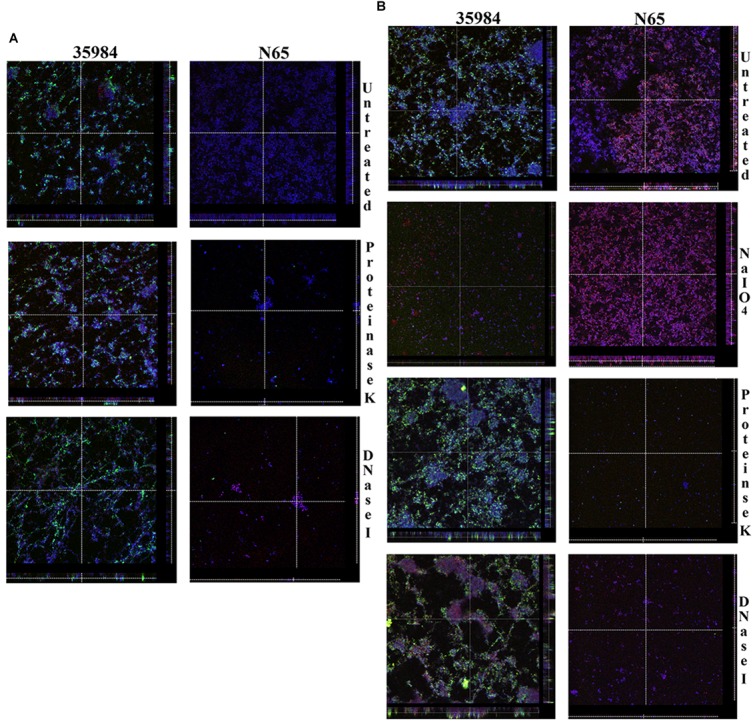
The figure shows the orthogonal views of CLSM images. **(A)** Biofilms formed by ATCC 35984 and SHN65 in the presence of proteinase K and DNase I, and **(B)** Detachment of preformed biofilms by adding NaIO_4_, proteinase K, and DNase I. Acquired the z-stacks of each chamber by CLSM with a Leica TCS SP5 confocal scanning system (Leica Microsystem, Mannheim, Germany) 63× oil objective lens.

Confocal laser scanning microscopy imaging of the biofilm formed by ATCC 35984 and SHN65 indicates that the ECM of ATCC 35984 is predominantly composed of polysaccharides which stained green in our assay; whereas, ECM of SHN65 which took both red and blue stains represent protein and DNA as principal components (Figure 3A). CLSM imaging of the detachment assay also inferred that ECM of ATCC 35984 contains polysaccharides and SHN65 contain protein and DNA (Figure 3B). The thickness of the biofilm formed by ATCC 35984 reduced upon NaIO_4_ treatment whereas no reduction was observed upon Proteinase K and DNase I treatment. On the contrary, the biofilm thickness of SHN65 decreased after treatment with proteinase K and DNase I and was unaffected by NaIO_4_ treatment (Figure 3B).

### Biofilm Inhibition Studies

Time point inhibition studies using proteinase K and DNase I determined the components of the biofilm matrix at different stages of biofilm development in *S. haemolyticus* strains SHN65 and SH1965. The addition of proteinase K (Supplementary Figures S4A,C) and DNase I Supplementary Figures S4B,D) at all time points diminished the cell adherence without showing any significant effect on bacterial growth.

### Temporal Effect of Autolysis on Biofilm Formation

**FIGURE 4:**
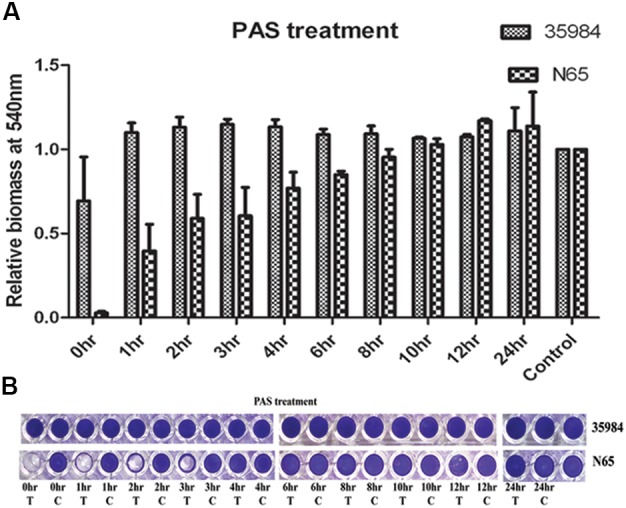
The effect of Sodium Polyanethole Sulphonate (PAS) on biofilm formation by *S. haemolyticus* strain SHN65 and *S. epidermidis* strain ATCC 35984 at different time points. **(A)** Normalized values of biofilm formed by strains after PAS treatment. **(B)** Images of wells of the microtiter plate stained with crystal violet. T: Treated, C: Control. Mean values from three independent experiments are shown, and error bars represent the SEM.

We used sodium PAS, a known chemical inhibitor of cell lysis which does not affect the growth of the bacteria at different time points of biofilm development. PAS added at 0–3 h of time points drastically reduced the biomass of the adherent cells of *S. haemolyticus* SHN65 (Figures 4A,B). However, no effect was observed on adherent biomass of positive control *S. epidermidis* ATCC 35984 (Figures 4A,B).

### PCR Assays

Although all the ocular isolates produced biofilm, none carried the *icaA* or *icaD* genes, irrespective of the sources of isolation. All isolates carried the genes encoding cell surface attachment proteins (*ebpS*, *cbp*, *fbp*, and *srtA*), quorum sensing (*agrA*, *agrB*, *agrD*, and *luxS*), eDNA release (*cidA*, *cidB*, *lrgA*, *lrgB*, *lytS*, *lytR*, and *atlE*), and global regulators (*sarA*, *mgrA*, and *sigB* and *araC/xylS*) (data not shown). These isolates also showed positive results for *mphC*, *ermC*, *msrA*, *aac6′-aph2″*, *ant4′*, *aph3′*, *tetK*, *dfrG*, *blaZ*, *mecA*, *cat:pC223*, *sat*, *lnuA* genes.

### RT-PCR Assays

**FIGURE 5:**
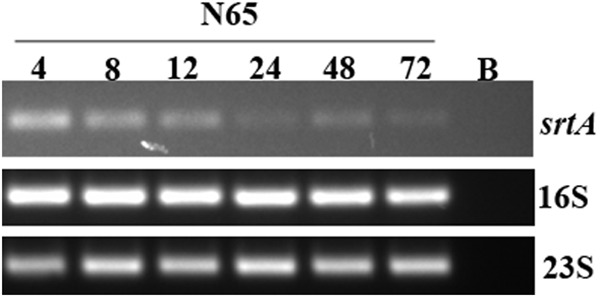
Results of RT-PCR obtained with *S. haemolyticus* strain SHN65 showing the expression of *srtA* at different time points in biofilm formation. Lane B. Negative control without template.

The expression study of biofilm-associated genes showed that majority isolates expressed genes associated with cell surface attachment, quorum sensing, eDNA release, and global regulation. We then correlated the expression of the genes associated with eDNA release and cell surface attachment proteins with the extent of detachment upon addition of DNase I and proteinase K, respectively. There was a good association between *cidA*, and *cidB* expression and detachment upon DNase I treatment. Similarly the expression of *fbp*, *ebpS*, *cbp*, and *srtA* genes showed an association with higher detachment upon proteinase K treatment among the strains (Supplementary Figures S5A,B). The expression of *atl* associated with higher detachment upon both DNase I and proteinase K treatment. We did not look for the association of expression of quorum sensing genes, and global regulators with biofilm component as these play a role in both eDNA and protein-rich biofilms. The expression of *srtA* was elevated at early time points of biofilm formation (Figure 5).

### PCR Assay of eDNA From Biofilm Matrix

The eDNA present in the mature biofilm of 18 ocular *S. haemolyticus* isolates and determined by RT-PCR is shown in Supplementary Figure S6A. In comparison to other strains, two of the isolates, SHN65 and SH314 produced more eDNA (Supplementary Figure S6B). Upon using eDNA as a template for PCR, these isolates showed positive results for *mphC*, *ermC*, *msrA*, *aac6′-aph2″*, *ant4*′, *aph3′*, *tetK*, *dfrG*, *blaZ*, *mecA*, *cat:pC223*, *sat*, *lnuA* genes (Supplementary Figure S7). These findings thus indicate that ARGs released in eDNA can act as a source for the transmission of ARGs.

## Discussion

Our study demonstrated that all ocular isolates of *S. haemolyticus*, from both infected eyes and asymptomatically healthy conjunctiva, were able to form biofilm. This observation is similar to those who also reported that both clinically significant and contaminant isolates of *S. haemolyticus* collected from human blood could form biofilm (Silva et al., 2013). One of the *S. haemolyticus* from healthy conjunctiva (SHN65) formed better biofilm and was comparable to that of the positive control strain of *S. epidermidis* ATCC 35984, but the composition of the matrix was different. We found that both TSB_glu_ and BHIB_glu_ are better media for the production of biofilm compared to that of TSB_NaCl_ and BHIB_NaCl_, except for one isolate (SH1965) that induced better biofilm in TSB_NaCl_. This finding is similar to those workers who also demonstrated that majority *S. haemolyticus* isolates produced better biofilm in TSB_glu_ than in TSB_NaCl_ (Fredheim et al., 2009; Barros et al., 2015). These observations thus suggest that TSB_glu_ and BHIB_glu_ are a better medium to study the biofilm development and inhibition studies.

We found that none of the strains showed >50% detachment when treated with NaIO_4_. Nine isolates showed >50% detachment upon treatment with proteinase K and six isolates with DNase I, respectively. However, four strains did not show any detachment upon NaIO_4_ or DNase I treatment. This finding thus suggests that either the protein or eDNA or both are the primary component of the biofilm matrix in most of the isolates. Moreover, NaIO_4_ induced biofilm disruption in 14 strains suggesting the presence of hitherto unknown β 1, 6-linked polysaccharides that could be part of the biofilm matrix, a finding similar to those reported by Fredheim et al. (2009). Analysis of CLSM images of best biofilm forming strain SHN65 showed a significant reduction in biomass upon treatment with proteinase K and DNase I. This observation thus indicates that the primary component of biofilm matrix consists of both protein and eDNA. The results of this study are similar to those workers who also demonstrated the functional relevance of proteins and eDNA in biofilm among *S. haemolyticus* (Beenken et al., 2004; Fredheim et al., 2009).

Polyanethole sulphonate is known to reduce the eDNA release and biofilm adherence in *S. aureus* (Rice et al., 2007). In this study, we observed that PAS within the first 4 h decreased the capacity of the biofilm to adhere on to the surface of microtiter wells. This step is a crucial step in biofilm development. However, the addition of PAS at the later time points did not have any effect on the biofilm development. This observation thus indicates that the accumulation of eDNA in the biofilm matrix during the early stages of biofilm formation is sufficient to allow the adherence throughout the process of biofilm development. The inhibition of biofilm formation by DNase I and proteinase K throughout the process indicate that DNA and proteins are the major components of ECM. Mann et al. (2009) also reported the inhibitory effect of PAS, DNase I and proteinase K on biofilm development in *S. aureus*.

Polysaccharide intercellular adhesin-dependent pathway has been described playing an essential role in biofilm development among *S. aureus* and *S. epidermidis* (Arciola et al., 2001; Gad et al., 2009). However, none of the isolates used in this study were positive for *icaA* or *icaD* genes indicating that the formation of biofilm in *S. haemolyticus* is PIA-independent. This observation corroborates the finding of other workers who also showed no role or least role of the *ica* operon in biofilm formation in *S. haemolyticus* isolates (Fredheim et al., 2009; Silva et al., 2013; Barros et al., 2015). The PIA-independent biofilm formation in other staphylococci such as *S. aureus*, *S. epidermidis*, *Staphylococcus lugdunensis* was also occasionally reported (Fitzpatrick et al., 2005; Frank and Patel, 2007; Qin et al., 2007). Based on the results of this study, we hypothesized that PIA-independent pathways might play a significant role in biofilm formation among ocular isolates of *S. haemolyticus*. Several studies have shown the role of the *ica* genes as virulence factors in staphylococcal infections (Rooijakkers et al., 2005; O’Gara, 2007) and also used as a tool to differentiate between virulent and non-virulent isolates (Namvar et al., 2013). In this study, we found all *S. haemolyticus* isolates, irrespective of sources of isolation, lack *icaA* and *icaD* genes indicating that there may be other factor(s) responsible for virulence in infected eye isolates. Also, the presence of *ica* genes cannot be used as marker to differentiate virulent and non-virulent isolates.

We then examined the presence of other biofilm-associated genes among *S. haemolyticus*. PCR results showed the presence of *srtA*, *atl*, *fbp*, *cbp*, and *ebpS* genes among all the isolates irrespective of the extent and type of biofilm formation by the isolates. These findings are similar to those workers who also reported the presence of *atl* and *fbp* genes in non-biofilm forming isolates of *S. haemolyticus* (Barros et al., 2015). Li et al. (2012) also reported the presence of genes encoding cell surface proteins such as fibrinogen binding protein, collagen binding protein and clumping factor in both biofilm-positive and -negative isolates of *S. aureus*. Several workers showed that *cidABC* and *lrgAB* operon, and the LytSR two-component system has a role to play in cell lysis and death, eDNA release and biofilm development in *S. aureus* (Rice et al., 2007; Ranjit et al., 2011). The presence of *cidABC*, *lrgAB*, and *LytSR* genes among all *S. haemolyticus* isolates indicate their possible role in biofilm formation. All the genes, alone or in combination, were reported playing roles in the formation of biofilm among *S. aureus* and *S. epidermidis* (Beenken et al., 2004; Rice et al., 2007; Periasamy et al., 2012). Also, *S. haemolyticus* strains used in the study carried quorum sensing genes (*agrA*, *agrB*, *agrD*, and *luxS*) and global regulators (*sarA*, *mgrA*, and *sigB*, and *araC/xylS*); therefore, their role in biofilm development cannot be ruled out in *S. haemolyticus* isolates.

However, the omnipresence of these biofilm associated genes among all isolates irrespective of the type of biofilm produced clearly indicates that the level of gene expression might play a role in dictating the extent and structure of biofilm. All ocular isolates, except one isolate, expressed *cidABC*, *lrgAB*, *LytSR*, *agrA*, *agrB*, *agrD*, and *luxS*, *sarA*, *mgrA*, and *sigB* and *araC/xylS* genes, 14 isolates showed expression of *atl*, and 16 strains showed expression of *mgrA* indicating their probable role in biofilm formation and development. We found a good correlation between expression of *cidA*, and *cidB* with eDNA component and *efb*, *ebp*, *cbp*, and *srtA* genes with protein component indicating their role in determining biofilm structure. However, mutational studies need to be performed to confirm the above finding. To our knowledge, this is the first report which depicts the presence and expression of *cidABC* and *lrgAB*, quorum sensing genes and global regulators among *S. haemolyticus* isolates. The abundance of *srtA* transcript during early time points thus suggests that Sortase A has a role to play during initial stages of the biofilm formation and development.

Several workers reported that eDNA is a structural component of biofilm matrix produced by *P. aeruginosa*, *S. aureus*, *S. epidermidis*, and *S. haemolyticus* (Rice et al., 2007; Fredheim et al., 2009). Also, in the present study, we found that a majority of *S. haemolyticus* isolates released DNA indicating that eDNA could be one of the major components of the biofilm matrix. Besides being a structural component of the biofilm matrix, eDNA is also demonstrated playing a pivotal role in mediating HGT in bacteria, e.g., *Haloferax volcanii* (Chimileski et al., 2014). To decipher the role of eDNA, if any, in HGT, we examined the presence of ARGs in eDNA of *S. haemolyticus*. The eDNA released by *S. haemolyticus* strains showed the presence of *mphC*, *ermC*, *msrA*, *aac6′-aph2″*, *ant4′*, *aph3′*, *tetK*, *dfrG*, *blaZ*, *mecA*, *cat:pC223*, *sat*, *lnuA* genes. The most prevalent ARGs in eDNA was *blaZ* followed by *mecA* and *aac6′-aph2″*. These ARGs were also amplified from the cell lysates used as a template from the respective *S. haemolyticus* isolates (Panda et al., 2016). The presence of ARGs in eDNA biofilm matrix thus suggests that eDNA might have a role to play in HGT; however, this phenomenon needs to be validated.

## Conclusion

In conclusion, this study shows that PIA-independent biofilm formation is a standard feature of *S. haemolyticus* isolates, irrespective of the sources of isolation. To our knowledge, this is the first report depicting the presence and expression of biofilm-associated genes, quorum sensing genes, and global regulators among *S. haemolyticus*. These finding further indicates that either the protein or DNA or both are the primary component of the biofilm matrix in most of the isolates. The presence of antibiotic resistance genes in eDNA suggests their possible role in HGT of antibiotic-resistant determinants. Cell lysis enabling DNA release was an essential step for biofilm attachment during the initial stages of biofilm development. The eDNA and protein remain a vital matrix component throughout the process of biofilm maturation. The expression of *srtA* transcript decreases with the biofilm age indicating its role in the early stages of biofilm development. Studies on the temporal and stochastic expression of biofilm-associated genes, the release of eDNA and composition of proteins in biofilm matrix are warranted to understand the mechanism involved in *S. haemolyticus* biofilm development.

## Author Contributions

SP and DVS conceived the experiments. SP conducted the experiments. SP and DVS analyzed the results. SP and DVS wrote the paper. All authors reviewed and approved the manuscript.

## Conflict of Interest Statement

The authors declare that the research was conducted in the absence of any commercial or financial relationships that could be construed as a potential conflict of interest.
