# Supplementary material for: Biofilm Formation by ica-Negative Ocular Isolates of Staphylococcus haemolyticus
Source: Front Microbiol. 2018 Nov 14;9:2687. doi: 10.3389/fmicb.2018.02687 (PMC6247817; doi:10.3389/fmicb.2018.02687)
Supplement: Supplementary file 1 [file Data_Sheet_1.pdf]

Table S1. Sequences of primers and cyclic conditions used for amplification of biofilm-associated and antibiotic resistance genes of 18 ocular isolates of *S. haemolyticus* used in this study.

Target	Primers	Sequence (5'-3')	Cyclic Conditions	Amplicon size (bp)	References
icaA	icaAF<br>icaAR	TCTCTTGCAGGAGCAATCAA<br>TCAGGCACTAACATCCAGCA	D at 94°C for 30″<br>A at 56°C for 30″ } 35 cycles<br>E at 72°C for 30	188	Arciola et al., 2001
icaD	icaDF<br>icaDR	ATGGTCAAGCCCAGACAGAG<br>CGTGTTTTCAACATTTAATGCAA	D at 94°C for 30″<br>A at 56°C for 30″ } 35 cycles<br>E at 72°C for 30	198	Arciola et al., 2001
sarA	sarAhaemo175F<br>sarAhaemo175R	AGCAATGATCACATACGCAGA<br>CACTGCTTTAACTACTTGTGGTTGT	D at 94°C for 30″<br>A at 52°C for 30″ } 30 cycles<br>E at 72°C for 30″	175	This study
lrgA	lrgAhaemoF<br>lrgAhaemoR	CCCTCCCCAAGTTTAGTTTGTT<br>ATTTCCTATGCCAGCGTCAGT	D at 94°C for 30″<br>A at 54°C for 30″ } 30 cycles<br>E at 72°C for 30″	315	This study
lrgB	lrgBhaemoF<br>lrgBhaemoR	GGTGGAACAATGGCAACGAT<br>ATGGTTGCGGGGATCGTATT	D at 94°C for 30″<br>A at 52°C for 30″ } 30 cycles<br>E at 72°C for 30″	551	This study
agrA	agrAhaemoF<br>agrAhaemoR	CCGAAGCAAAACAGTGCTCA<br>AGATTGCGCTTGCAACAGAC	D at 94°C for 30″<br>A at 54°C for 30″ } 30 cycles<br>E at 72°C for 30″	600	This study
agrB	agrBhaemoF<br>agrBhaemoR	GCTTAAACGGTTCTGGTGCT<br>ACACGGTGCACATGCAAAAT	D at 94°C for 30″<br>A at 54°C for 30″ } 30 cycles<br>E at 72°C for 30″	276	This study

agrC	agrChaemo183F<br>agrChaemo183R	ACGTGCGATTAAAGGCTTGG<br>AACGCATCTTCAAGTGCTTCAG	D at 94°C for 30″<br>A at 52°C for 30″ } 30 cycles<br>E at 72°C for 30″	183	This study
sigB	sigBhaemoF<br>sigBhaemoR	GTACCGACCGTTATTGGTGAGA<br>GTTCCCCAGTTTCTTTCTGGC	D at 94°C for 30″<br>A at 52°C for 30″ } 30 cycles<br>E at 72°C for 30″	430	This study
luxS	luxShaemoF<br>luxShaemoR	GCCCAACCGCATTGTACTTC<br>GTTGTTGCACCTTTCGTCCG	D at 94°C for 30″<br>A at 54°C for 30″ } 30 cycles<br>E at 72°C for 30″	332	This study
ebpS	EbpShaemoF<br>EbpShaemoR	TTCCCACCTAGAAACGCACA<br>TTAGCGTCACGATGGTCGTC	D at 94°C for 30″<br>A at 55°C for 30″ } 30 cycles<br>E at 72°C for 30″	605	This study
fbp	Fbphaemo292F<br>Fbphaemo292R	CCACCAATGTTTGCTCGTGT<br>TGTTTTGAGTAGGTGGCGCT	D at 94°C for 30″<br>A at 55°C for 30″ } 30 cycles<br>E at 72°C for 30″	292	This study
cbp	Cbphaemo383F<br>Cbphaemo383R	TACGCTAGCGCAAGGTGAAT<br>TGTTTAACGGACGGCCAACT	D at 94°C for 30″<br>A at 55°C for 30″ } 30 cycles<br>E at 72°C for 30″	383	This study
atl	atlhaemo594F<br>atlhaemo594R	GCAAAGGTGGTCCTGCTACT<br>GGTAAGCGTGTGGGTCAGAA	D at 94°C for 30″<br>A at 55°C for 30″ } 30 cycles<br>E at 72°C for 45″	594	This study
cidA	cidAhaemoF<br>cidAhaemoR	CTAGCTGGAAGTATCGTAGGCA<br>ACCATYTTTTCAGCGATGTAACC	D at 94°C for 30″<br>A at 52°C for 30″ } 30 cycles<br>E at 72°C for 30″	251	This study
cidB	cidBhaemoF<br>cidBhaemoR	CCCAGCGTTAGTAGGTTCGG<br>TTTGCGGTACCRAAAGCGTG	D at 94°C for 30″<br>A at 52°C for 30″ } 30 cycles<br>E at 72°C for 30″	480	This study

srtA	srtAhaemoF<br>srtAhaemoR	AAACAAACACCAACCATCCCT<br>CCCAAACACCGGTTTGTTCAT	D at 94°C for 30″<br>A at 54°C for 30″ } 30 cycles<br>E at 72°C for 30″	406	This study
mgrA	mgrAhaemoF<br>mgrAhaemoR	TGCACAAAGACAAGTTAATCGC<br>AGAAGAAGCATTCGCGACTTT	D at 94°C for 30″<br>A at 52°C for 30″ } 30 cycles<br>E at 72°C for 30″	313	This study
lytS	LytShaemoF<br>LytShaemoR	TGTATCAGGGTTGTCACGCC<br>CGTACGAGGGCTGAAATGGT	D at 94°C for 30″<br>A at 55°C for 30″ } 30 cycles<br>E at 72°C for 30″	891	This study
lytR	LytRhaemoF<br>LytRhaemoR	TTTTTGCAACGGCACACGAT<br>TCTCCCGATGTCATGGTTACT	D at 94°C for 30″<br>A at 52°C for 30″ } 30 cycles<br>E at 72°C for 30″	478	This study
arcA/xyl	Arca/XylShaemo361F<br>Arca/XylShaemo361R	CGGAAAAGAAGGGGCCATTG<br>TCGGTACTCGTTGGGAGTCA	D at 94°C for 30″<br>A at 55°C for 30″ } 30 cycles<br>E at 72°C for 30″	361	This study
cidA	cidAhaemoRT F<br>cidAhaemoRT R	ATTGGATATCTGACGGCGCA<br>GCTAGTGCAACGCAACTTGT	D at 94°C for 30″<br>A at 52°C for 30″ } 25 cycles<br>E at 72°C for 30″	157	This study
cidB	cidBhaemoRT F<br>cidBhaemoRT R	CAGCTGCTGTAGGTATCGAAGT<br>CCCATATGTTAACCCTCTGGCA		161	This study
lrgA	lrgAhaemoRT F<br>lrgAhaemoRTR	CCCTCCCCAAGTTTAGTTTG<br>ATTCGTACCGGCCGGAATTT		192	This study
lrgB	lrgBhaemoRT F<br>lrgBhaemoRT R	AACGGCGATACTACCCATTG<br>CCCTTCCAGTATCACAAGGT		227	This study
sigB	sigBhaemoRT F<br>sigBhaemoRT R	CCGACCGTTATTGGTGAGAT<br>ACGGTCAGCAATTTCTGC		162	This study
lytS	lytShaemoRT F	CTGGTTTAATTGGCGGACCT		187	This study

	lytShaemoRT R	GGCACCCATTAAAACGGTTG			
lytR	lytRhaemoRT F<br>lytRhaemoRT R	CCACTCATCGCACATATC<br>CTCCCGATGTCATGGTTACT		168	This study
agrA	agrAhaemoRT F<br>agrAhaemoRT R	GAAACTGCACACACTCGCTT<br>AACCGCTCATCAAGTTGTGC	D at 94°C for 30″<br>A at 52°C for 30″ } 25 cycles<br>E at 72°C for 30″	215	This study
agrB	agrBhaemoRT F<br>agrBhaemoRT R	GTTGCTGAAGGAGCATAG<br>TTATGCACACGGTGCACATGCA		171	This study
agrC	agrChaemoRT F<br>agrChaemoRT R	CGACTAAGGTCAATCGTG<br>CGAGAAGATGATATGCCAGG		236	This study
luxS	luxShaemoRT F<br>luxShaemoRT R	AATGACTTGCTGCCCAACCGCATT<br>GGGTTACACTCACTAGAGCA		229	This study
atl	atlhaemoRT F<br>atlhaemoRT R	CAACATGCACATGCAGCTGA<br>GAAGTCTGTTGTGTTGTCGC		236	This study
srtA	srtAhaemoRT F<br>srtAhaemoRT R	CACCAACCATCCCTAAGGAT<br>GGTAATGTGGTCTATCGG		201	This study
araC/xylS	AraC/XylShaemoRT F<br>AraC/XylShaemoRT R	CGCTAACGGGTATAGCATCGAA<br>TCGTTGGGAGTCATGTCAAC		238	This study
ebpS	ebpShaemoRT F<br>ebpShaemoRT R	AAGACGCTGCAATAGCTGGA<br>ACGATGGTCGTCATGAAGGT		191	This study
fbp	fbphaemoRT F<br>fbphaemoRT R	CGCGTTAAACAACGCGCAAA<br>ACAGTTACAGCGTCATCACC		188	This study
cbp	cbphaemoRT F<br>cbphaemoRT R	CACGGCTTCCAATCCACAAT<br>CAATGGCTTGCGCATTTGCA		194	This study
gyrA	gyrAhaemoRTF<br>gyrAhaemoRTR	GAAAGAGAGCCGGAAGTCTT<br>GTCGGATGCCACTTTTCCCTAGAA	D at 94°C for 30″<br>A at 52°C for 30″ } 25 cycles<br>E at 72°C for 30″	235	This study
16S	16ShaemoRT F	GCGTGCCTAATACATGCAAGTCGA		203	This study

	16ShaemoRT R	GCGGGTCCATCTATAAGTGATAGC			
23S	23ShaemoRT F<br>23ShaemoRT R	GGAAGGGGAGTGAAAGAGAACTTG<br>ATGGGTAGATCACCTGGTTTCG		206	This study
blaZ	blaZF<br>blaZR	ACTTCAACACCTGCTGCTTTC<br>TGACCACTTTTATCAGCAACC	D at 94°C for 30″<br>A at 52°C for 30″<br>E at 72°C for 30″ } 25cycles	173	Argudín, et al., 2011
mphC	mphCF<br>mphcR	TTACAGGTAAACCCGCAGCC<br>CATTGATGGGTCGGAGTGGG	D at 94°C for 30″<br>A at 58°C for 30″<br>E at 72°C for 30″ } 25cycles	407	This study
msrA	msrAF<br>msrAR	GCAAATGGTGTAGGTAAGACAACT<br>ATCATGTGATGTAAACAAAAT	D at 94°C for 30″<br>A at 45°C for 30″<br>E at 72°C for 30″ } 30 cycles	400	Duran et al. 2012
ermA	ermAF<br>ermAR	GCGGTAAACCCCTCTGAG<br>GCCTGTCGGAATTGG	D at 94°C for 30″<br>A at 50°C for 30″<br>E at 72°C for 30″ } 30 cycles	434	Schlegelova, et al., 2008
ermC	ermCF<br>ermCR	ATCTTTGAAATCGGCTCAGG<br>CAAACCCGTATTCCACGATT	D at 94°C for 30″<br>A at 62°C for 30″<br>E at 72°C for 30″ } 25 cycles	295	Schlegelova, et al., 2008
lnuA	lunAF<br>lnuAR	GGTGGCTGGGGGGTAGATGTATTAACTGG<br>GCTTCTTTTGAAATACATGGTATTTTTCGATC	D at 94°C for 30″<br>A at 55°C for 30″<br>E at 72°C for 30″ } 25 cycles	323	Lozano et al., 2012
aac6´-aph2″	aac6´-aph2″F<br>aac6´-aph2″R	CCAAGAGCAATAAGGGCATACC<br>CACACTATCATAACCATCACCG	D at 94°C for 30″<br>A at 52°C for 30″<br>E at 72°C for 30″ } 25 cycles	348	Schmitz et al., 1999
ant4′	ant4′F<br>ant4′R	CTGCTAAATCGGTAGAAGC<br>CAGACCAATCAACATGGCACC	D at 94°C for 30″<br>A at 62°C for 30″<br>E at 72°C for 30″ } 25 cycles	173	Schmitz et al., 1999

aph3′	aph3′F<br>aph3′R	CTGATCGAAAAATACCGCTGC<br>TCATACTCTTCCGAGCAAAGG	D at 94°C for 30″<br>A at 60°C for 30″ } 25 cycles<br>E at 72°C for 30″	269	Schmitz et al., 1999
tetK	tetKF<br>tetKR	GGTTTGTTTTCTGGTGGGCT<br>GCAAACTCATTCCAGAAGCAACT	D at 94°C for 30″<br>A at 58°C for 30″ } 25cycles<br>E at 72°C for 30″	385	This study
dfrG	dfrGF<br>dfrGR	TGCTGCGATGGATAAGAA<br>TGGGCAAATACCTCATTCC	D at 94°C for 30″<br>A at 55°C for 30″ } 25 cycles<br>E at 72°C for 30″	405	Panda et al., 2016
dfrA	dfrAF<br>dfrAR	CACTTGTAATGGCACGGAAA<br>CGAATGTGTATGGTGGAAAG	D at 94°C for 30″<br>A at 50°C for 30″ } 30 cycles<br>E at 72°C for 30″	270	Panda et al., 2016
*cat:*pC223	cat:pC223F<br>cat:pC223R	AGGATATGAACTGTATCCTGCTTTG<br>AATAATGAAACATGGTAACCATCAC	D at 94°C for 30″<br>A at 52°C for 30″ } 20 cycles<br>E at 72°C for 30″	464	Panda et al., 2016
sat	satF<br>satR	AAAGCAGGGCACCTGAAAGA<br>TATGCCTTGCCCCCTGAAAT	D at 94°C for 30″<br>A at 57°C for 30″ } 20 cycles<br>E at 72°C for 30″	324	This study

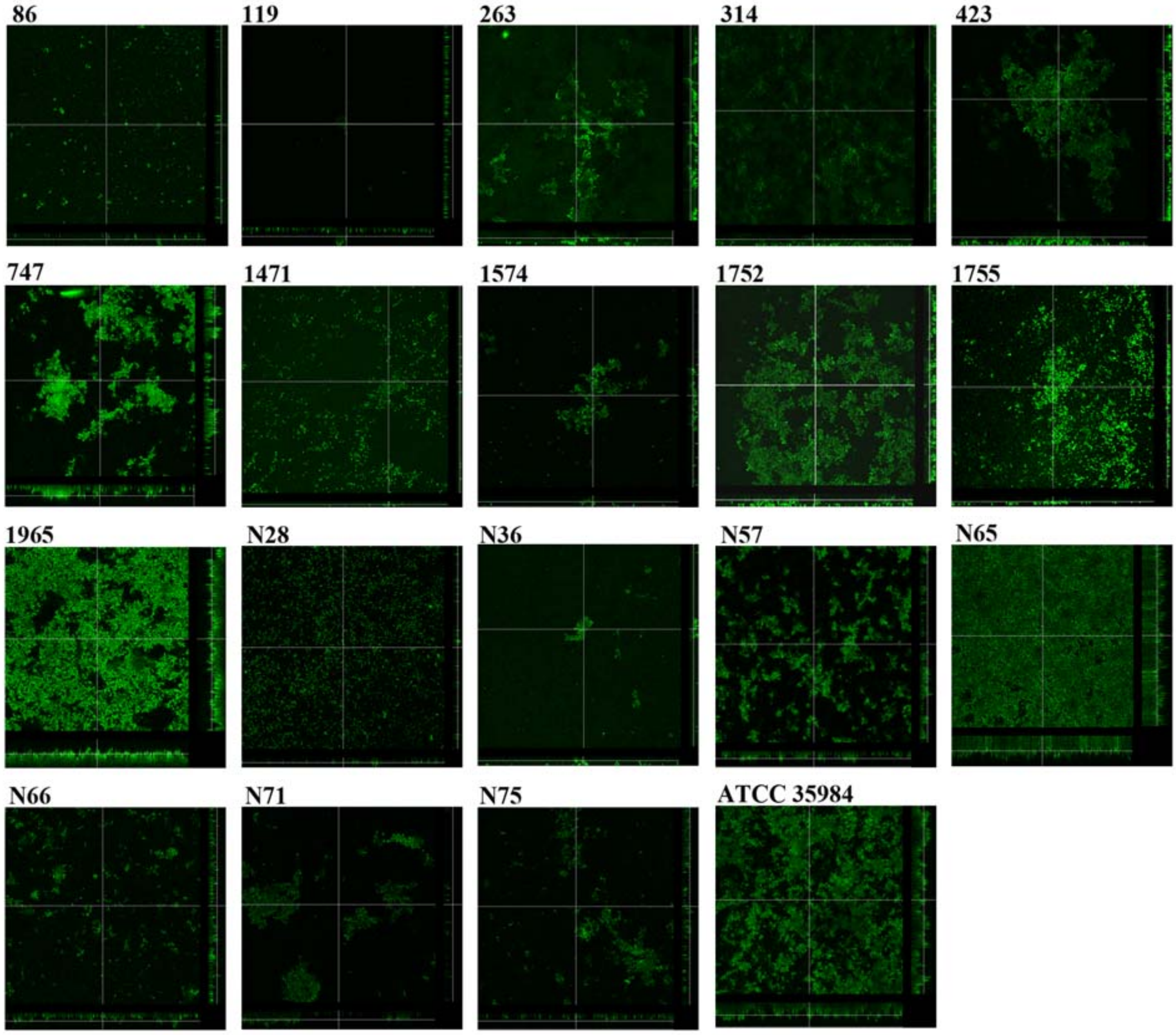

Fig. S1. Orthogonal view of CLSM images of static biofilm of 18 ocular isolates of *S. haemolyticus* and *S. epidermidis* ATCC 35984. All the strains were grown in TSB_{glu} as a static biofilm. After 24 hrs growth, washed the biofilms and stained with Acridine Orange, and CLSM acquired z-stacks of each chamber at 1 μm interval with a Leica TCS SP5 confocal scanning system (Leica Microsystem, Mannheim, Germany) using 63X oil objective lens.

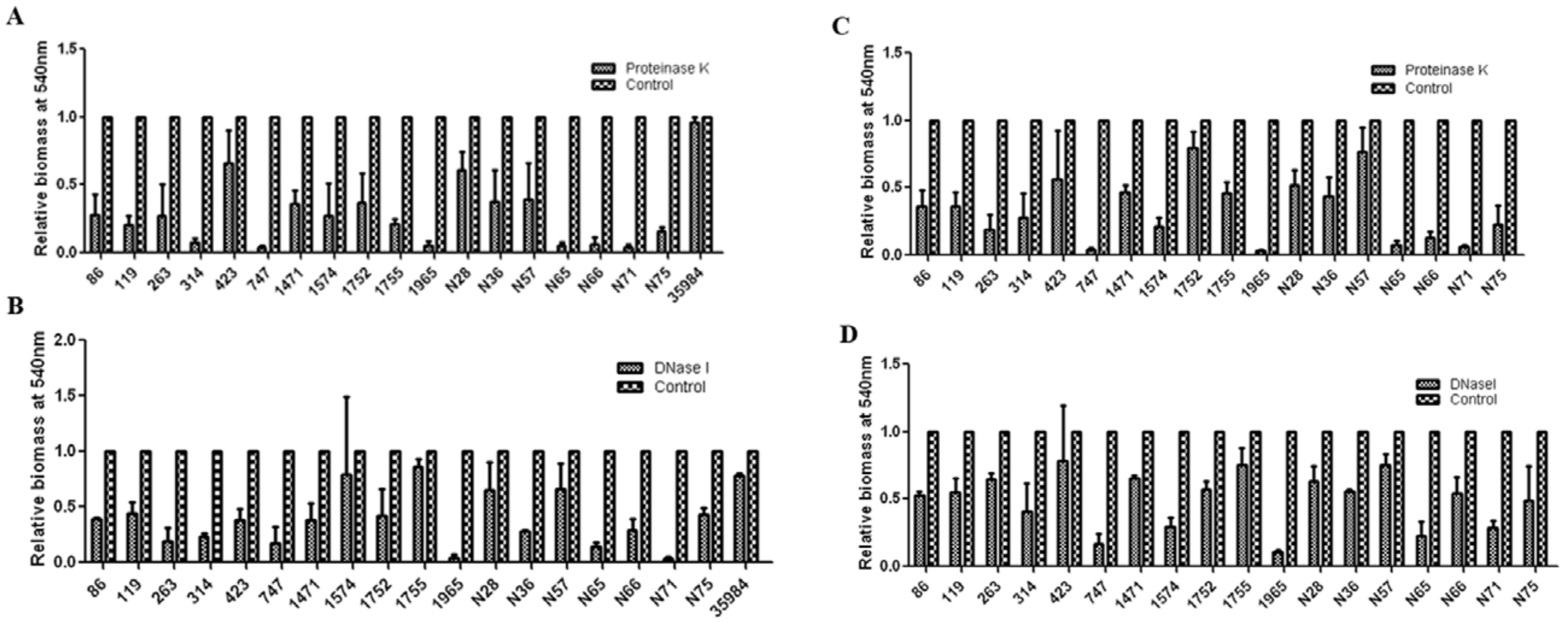

Fig. S2. Results of inhibition studies on biofilm formation in TSB_{glu} **(A, B)** and $BHIB_{glu}$ **(C, D)** obtained with 18 ocular *S. haemolyticus* isolates with Proteinase K **(A, C)** and DNase I **(B, D)**. Mean values from three independent experiments are shown and error bars represent the standard error mean.

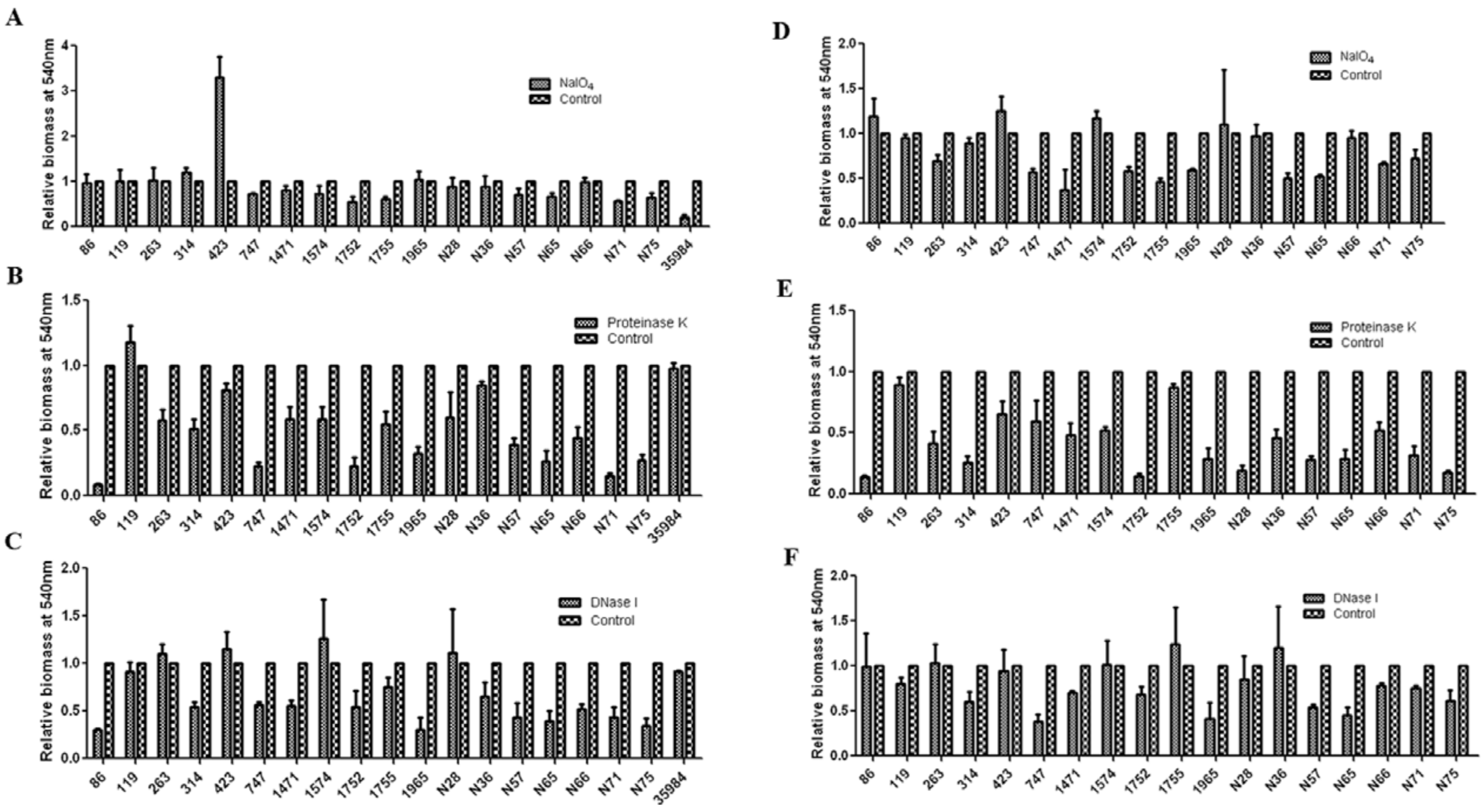

Fig. S3. Results of biofilm detachment assay obtained after treatment of preformed biofilms grown in TSB_{glu} **(A-C)**, and $BHIB_{glu}$ **(D-F)**. Mean values from three independent experiments are shown and error bars represent the standard mean errors.

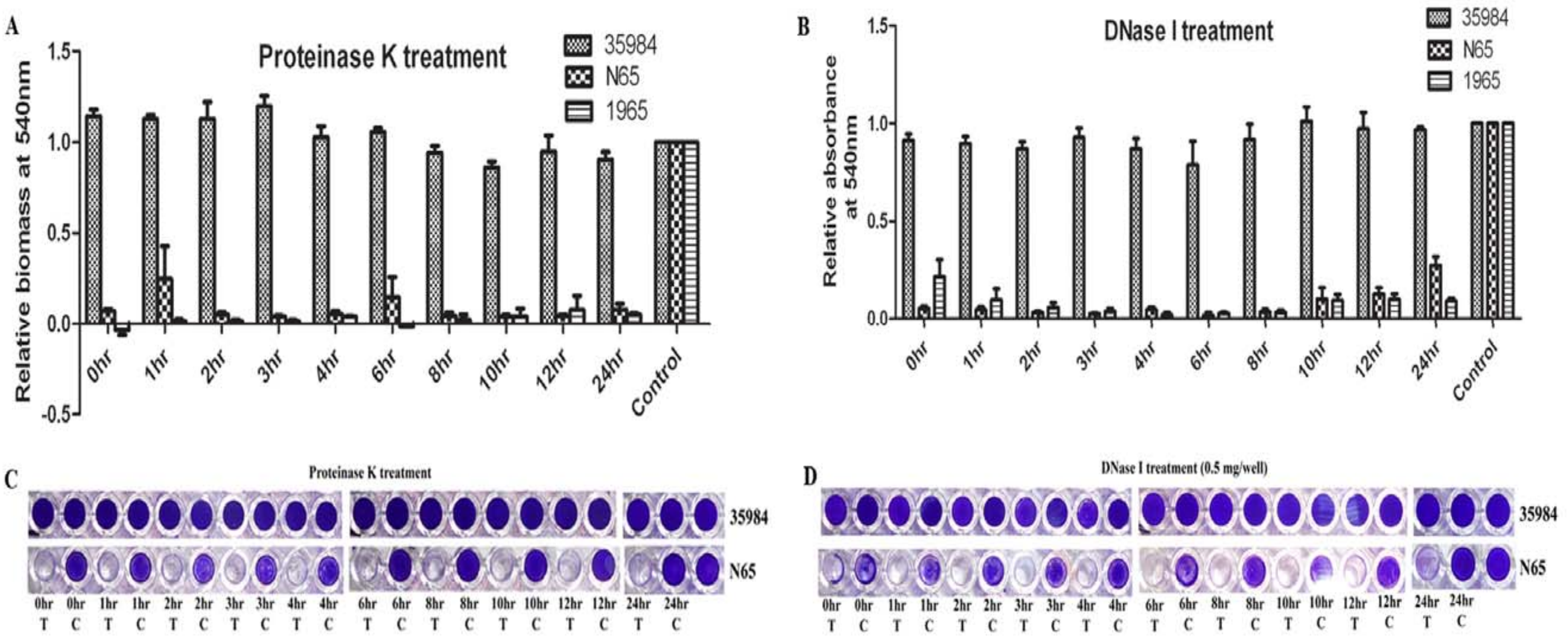

Fig. S4. The inhibition of biofilm formed by *S. haemolyticus* SHN65, SH1965 and *S. epidermidis* ATCC 35984 with proteinase K **(A)** normalized value of biofilm and **(C)** images of wells of the microtiter plate stained with crystal violet, and DNase I **(B)** normalized value of biofilm and **(D)** images of wells of the microtiter plate stained with crystal violet, at different time points.

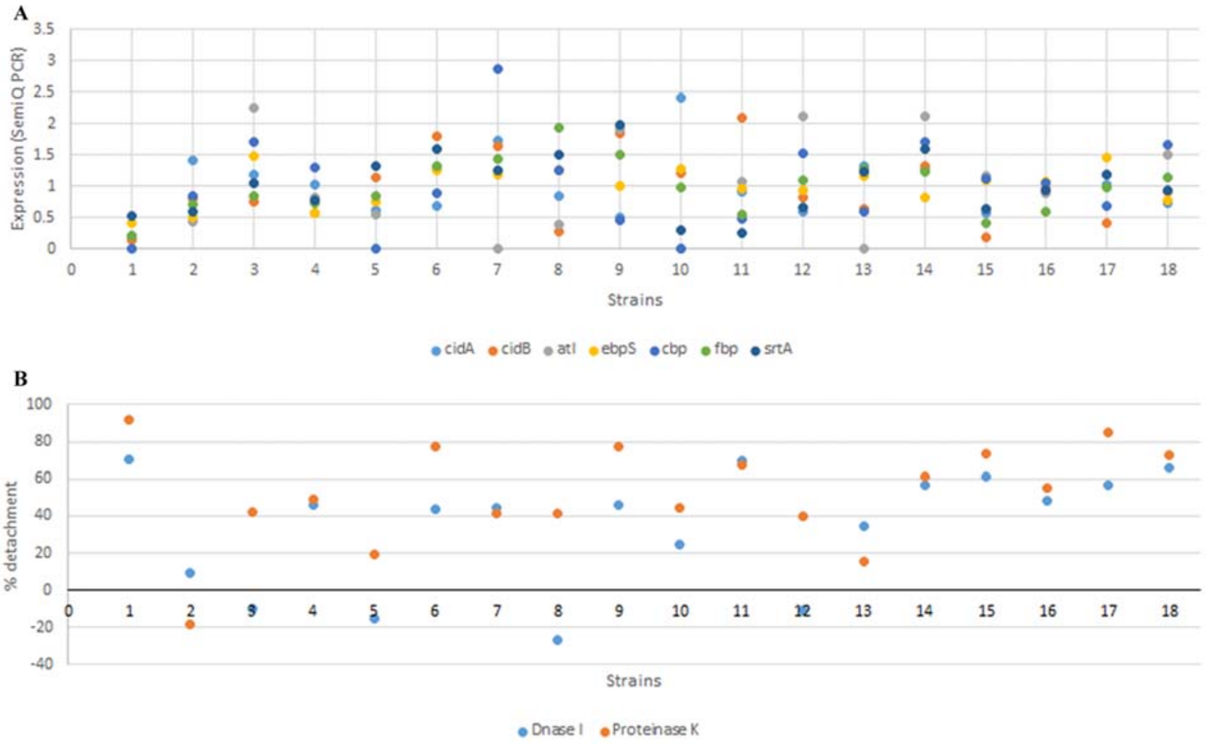

Fig. S5 (A) Results of expression of biofilm associated genes from static biofilms (grown for 4hrs) of 18 ocular *S. haemolyticus* isolates. **(B)** Results of detachment assay of pre-formed static biofilms (grown for 24 hrs) of 18 ocular *S. haemolyticus* isolates upon DNase I and proteinase K treatment. Strain 1, SH86; Strain 2, SH119; Strain 3, SH263; Strain 4, SH314; Strain 5, SH423; Strain 6, SH747; Strain 7, SH1471; Strain 8, SH1574; Strain 9, SH1752; Strain 10, SH1755; Strain 11, SH1965; Strain 12, SHN28; Strain 13, SHN36; Strain 14, SHN57; Strain 15, SHN65; Strain 16, SHN66; Strain 17, SHN71; Strain 18, SHN75.

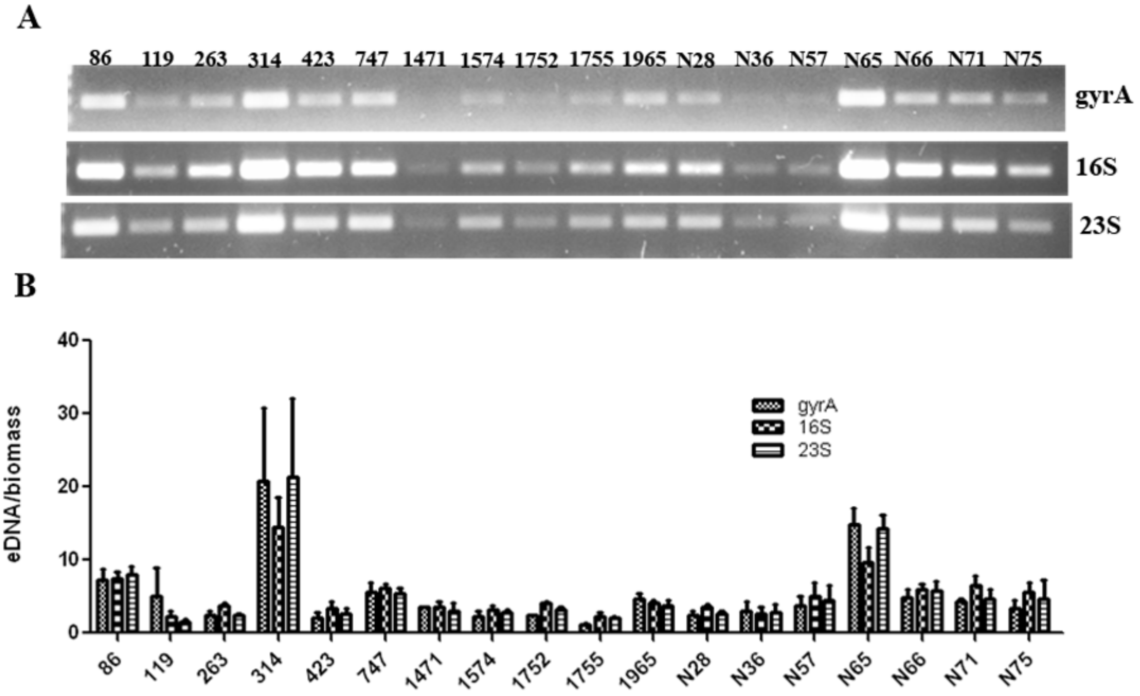

Fig. S6. The eDNA present in the biofilm matrix of *S. haemolyticus* isolates was quantified using *gyrA*, 16S, and 23S RNA genes. **(A)** Agarose gel electrophoresis of RT-PCR products obtained with *S. haemolyticus* isolates. **(B)** Densitometry analysis of the RT-PCR products obtained with *S. haemolyticus* isolates. The data are mean values from three independent experiments, and error bars represent the SEM.

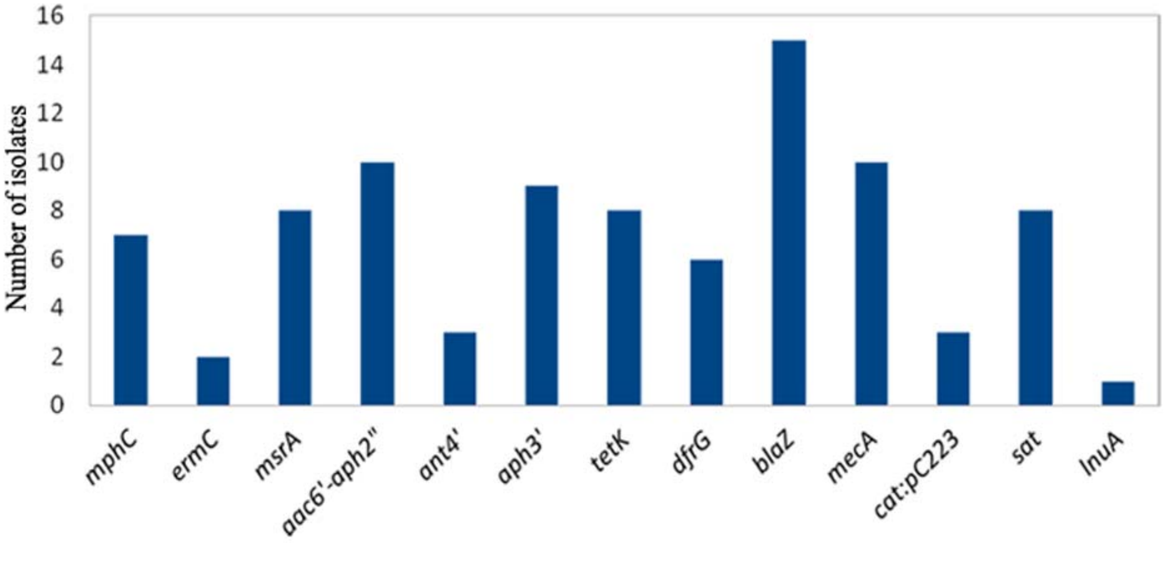

Fig. S7. Prevalence of various ARGs found in eDNA of 18 ocular *S. haemolyticus* isolates. *mphC*: streptogramin, *ermC:* erythromycin, *msrA*: macrolide*; aac6'-aph2", ant4',* and *aph3'*: aminoglycosides; *tetK*: tetracycline; *blaZ*: β-lactamase; *mecA*: methicillin; *cat: p233*: chloramphenicol; *sat*: streptothricin; *InuA*: lincosamide.

References

Arciola, C. R., Baldassarri, L., and Montanaro, L. (2001). Presence of *icaA* and *icaD* genes and slime production in a collection of staphylococcal strains from catheter-associated infections. *J. Clin. Microbiol.* 39, 2151-2156.

Argudín, M. A., Tenhagen, B. A., Fetsch, A., Sachsenröder, J., Käsbohrer, A., Schroeter, A., et al. (2011). Virulence and resistance determinants of German *Staphylococcus aureus*ST398 isolates from nonhuman sources. *Appl. Environ. Microbiol.* 77, 3052-3060.

Duran. N., Ozer, B., Duran, G. G., Onlen, Y., and Demir, C. (2012). Antibiotic resistance genes and susceptibility patterns in staphylococci. *Indian J. Med. Res.* 135, 389–396.

Lozano, C., Aspiroz, C., Sáenz, Y., Ruiz-García, M., Royo-García, G., Go´mez-Sanz, E., et al. (2012). Genetic environment and location of the *lnu(A)* and *lnu(B)* genes in methicillin-resistant *Staphylococcus aureus* and other staphylococci of animal and human origin. *J. Antimicrob. Chemother.* 67, 2804–2808.

Panda, S., Kar, S., Sharma, S., and Singh, D. V. (2016). Multidrug-resistant *Staphylococcus haemolyticus* isolates from the infected eye and healthy conjunctiva. *J. Glob. Antimicrob. Resist.* 6, 154-159. doi: 10.1016/j.jgar.2016.05.006.

Schlegelova, J., Vlkova, H., Babak, V., Holasova, M., Jaglic, Z., Stosova, T., Sauer, P. (2008). Resistance to erythromycin of *Staphylococcus* spp. isolates from the food chain. *Vet. Med.* 53, 307–314.

Schmitz, F. J., Fluit, A. C., Gondolf, M., Beyrau, R., Lindenlauf, E., Verhoef, J., et al. (1999). The prevalence of aminoglycoside resistance and corresponding resistance genes in clinical isolates of staphylococci from 19 European hospitals. *J. Antimicrob. Chemother.* 43, 253–259.
